# Case Report: Refractory Hypotension of GABA B Receptor Autoimmune Encephalitis

**DOI:** 10.3389/fneur.2020.571382

**Published:** 2020-12-01

**Authors:** Kong Hanxin, Wang Xiaomin, Ying Hao, Zheng Shuihong

**Affiliations:** ^1^Jinhua Central Hospital, Jinhua, China; ^2^Yuhang First People's Hospital, Hangzhou, China

**Keywords:** GABA B receptor autoimmune encephalitis, refractory hypotension, autonomic nerve function, autoimmune disease, case report

## Abstract

**Introduction:** With the development of antibody detection technology, Gamma-Aminobutyric Acid (GABA) B receptor encephalitis is a known autoimmune disease. This paper describes a patient with refractory hypotension who suffered GABA B receptor autoimmune encephalitis.

**Case Report:** We describe a 63-year-old man with GABA B receptor autoimmune encephalitis who had hypotension on day 17 of the disease onset. Despite two rounds of immunoglobulin administration, high-dose intravenous steroid injections and immunosuppressive therapy on day 35 of hospitalization, psychiatric symptoms and seizures were significantly improved; however, the patient's blood pressure remained low.

**Conclusion:** This case study and literature review investigated the impairment of autonomic nerve function and its subsequent management in patients with GABA B receptor autoimmune encephalitis.

## Introduction

GABA B receptor encephalitis is a potentially treatable disorder characterized by psychomotor abnormalities, seizures and recent memory disorders (1). Most Han Chinese patients with GABA B receptor encephalitis had significant refractory epilepsy and improved nervous system after immunotherapy (2). Small-cell lung cancer is found in about one-third of patients who undergo chest computed tomography (CT) or Positron Emission Tomography (PET) (1). GABA B receptors are highly distributed in the brain and spinal cord, with the highest distribution found in the hippocampus, thalamus and cerebellum (3). Therefore, cerebrospinal fluid (CSF) analysis plays a central role in all diagnostic criteria for autoantibodies in patients with GABA B receptor encephalitis. Magnetic resonance imaging (MRI) and electroencephalogram (EEG) revealed that bilateral or unilateral hippocampus, amygdala and temporal lobe were the main brain regions implicated (1). As the disease gains more recognition, more symptoms are likely to be identified.

At present, there is no consensus on the treatment of GABA B receptor encephalitis, mainly referring to anti-N-methyl D-aspartate Receptor (anti-NMDAR) encephalitis, including immunotherapy and symptomatic support therapy. The first-line treatment for this disease includes immunotherapeutic drugs such as glucocorticoids, plasma exchange and intravenous immunoglobulin (IVIg) (4). The second-line treatment which includes rituximab and cyclophosphamide are mainly administered to patients with poor response to the first-line immunotherapeutic treatment (4). However, there is very little information about the treatment and outcome of refractory epileptic seizures and autonomic nervous dysfunction in patients with GABA B receptor encephalitis. This case report describes a patient with GABA B receptor autoimmune encephalitis who was hospitalized due to refractory epilepsy and psychiatric symptoms and development of intractable hypotension on day 13 after admission.

## Case Report

A 63-year-old male Asian with no prior medical history was admitted to a local hospital due to loss of consciousness, physical convulsions and psychiatric symptoms. The patient was discharged from the hospital after receiving an antiepileptic therapy. The patient returned home with progressive mood changes, restlessness, confusion, recurrent seizures and was admitted in our hospital. The other neurological tests carried out on the patient were normal. EEG examination showed no abnormality; head CT was negative and MRI control showed no structural changes. As at this time, the patient neither had abnormalities in the blood test indices nor signs of infection such as elevated body temperature. Initially, we suspected viral encephalitis; therefore, we administered acyclovir (0.5 g) every 8 h as an antiviral therapy; valproate (0.5 g) twice a day as an antiepileptic and olanzapine (5 mg) once a day to improve the mental symptoms.

**Figure 1:**
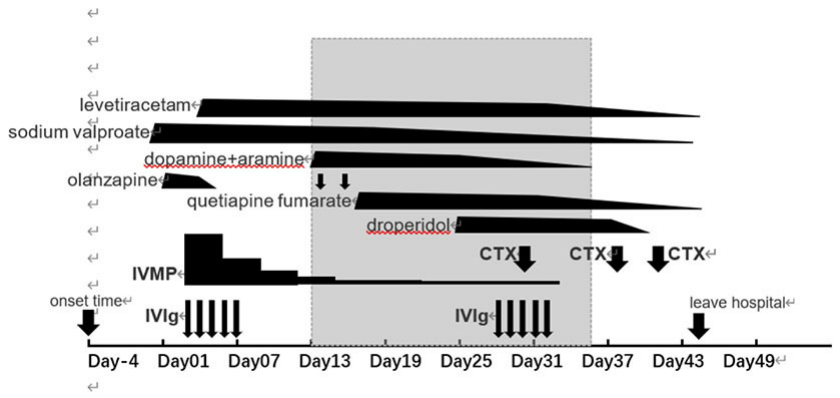
The clinical course. Gray bands indicate the length of time a patient has had intractable hypotension. IVIg, intravenous immunoglobulin; IVMP, intravenous methylprednisolone; CTX, cyclophosphamide.

On the second day of hospitalization, we performed lumbar puncture under local anesthesia and measured CSF pressure at 135 mm H_2_O. Initial CSF studies reported an elevated WBC count, which was dominated by lymphocytes (lymphocytes 86%, lactate dehydrogenase <100.0 μL, protein 406 mg/L, chloride 121 mmol/L, and glucose 3.02 mmol/L). There were 36 nuclear cells/μL in CSF, 4% lobules and 96% mononuclear cells. Cryptococcus neoformans was not found in CSF. Therefore, we ruled out the suspicion of tumor and infectious encephalitis. The positive autoantibody for anti-GABA B receptor neurons (titer IgG+++1:32) in CSF was detected by cellular immunoassay, thus confirming the diagnosis of anti-GABA B receptor encephalitis. Therefore, we stopped the empirical treatment of acyclovir and began a 5-day treatment course with IVIg immunoglobulin (2 g/kg/5 days) combined with daily 1,000 mg intravenous methylprednisolone shock therapy (Figure 1). After the 1,000 mg intravenous methylprednisolone shock therapy was administered for 3 consecutive days, the dosage was reduced to 500 mg once per day and then decreased once per 3 days. As a result of repeated seizures, the patient was administered up to 500 mg of levetiracetam twice per day. Following this, the patient developed an elevated body temperature and hypodermic oxygen. CT pulmonary angiography indicated pulmonary embolism; therefore, we administered the therapeutic dose of low molecular weight heparin. As at this time, the blood test indices indicated a leucocyte concentration of 19.19 × 10^9^/L, which comprised mainly increased neutrophils (16.91 × 10^9^/L), hypersensitive C-reactive protein (63.9 mg/L) and procalcitonin (1.088 ng/mL). Due to the indication of gram-negative bacterial infection by the patient's blood culture, piperacillin tazobactam sodium was administered as an anti-infection treatment. During this period, the patient's dynamic holter monitoring suggested sinus rhythm and head CT indicated patchy low-density lesions in the left hippocampus.

On day 13 of admission, the patient developed intractable hypotension. Blood pressure fluctuated between 70–90 and 46–83 mmHg. In this regard, our first suspicion was hypotension caused by the drug; therefore, we added 1,000 mL sugar saline to dilate the volume and discontinued some of the antihypertensive drugs. After a fruitless increase in fluid intake, we treated blood pressure with dopamine and aramine 2 days later. At day 18, we adjusted the dosage of dopamine, but the patient's blood pressure was still low. During this period, we also suspected hypotension caused by septic shock, but at this time, the patient's body temperature and heart rate were stabilized; serum hypersensitive C-reactive protein was 4 mg/L, white blood cell was 9.56 × 10^9^/L, among which the neutrophils was 6.95 × 10^9^/L. Combined with the obvious abnormalities of liver and kidney function and absence of obvious petechiae and other peripheral signs that indicate low perfusion of tissues and organs, we excluded the suspicion of persistent hypotension caused by sepsis infection. After discussion with a cardiovascular specialist, the patient was suspected to have intractable hypotension due to autonomic dysfunction caused by anti-GABA B receptor encephalitis. At this point, the patient presented symptoms of paroxysmal irritability and hallucinations, which were obvious at night, but without seizure. To control the mental symptoms, we administered quetiapine fumarate (0.2 g) per night. After this, the patient still had recurrent episodes of mental symptoms, sometimes intense irritability and even attempted to sit up in bed with restraint. As a result, we administered 5 mg of haloperidol intramuscular injection when necessary.

On day 28 of admission, the patient was able to respond partially. The fever was reduced than previous, but the patient still had recurrent psychiatric symptoms and autonomic nervous dysfunction, especially intractable hypotension. Owing to this, the patient must be continuously pumped with dopamine and serotonin to maintain a stable blood pressure. In consideration of the patient's prognosis, a second 5-day course of IVIg immunoglobulin therapy was initiated. After end of the immunoglobulin treatment, we initiated a second-line treatment with the immunosuppressant cyclophosphamide (CTX) (0.4 g) once per week and 60 mg of methylprednisolone once per day. Following the first immunosuppressive treatment, the patient's blood pressure did not improve significantly.

**Figure 2:**
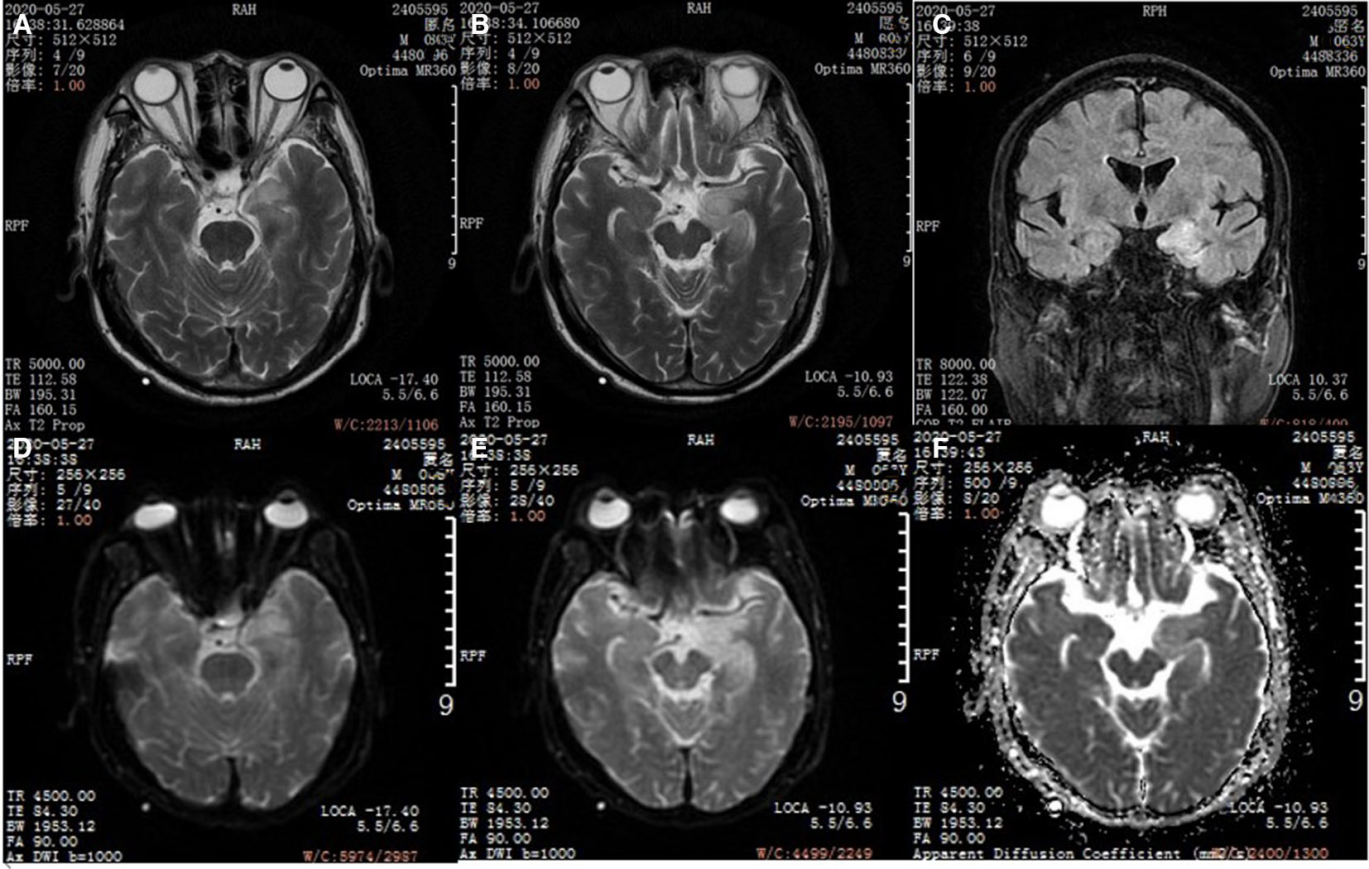
Head magnetic resonance imaging (MRI). **(A,B)** T2WI axial image in a patient with anti-GABA B encephalitis. **(C)** FLAIR corona scanning. **(D,E)** DWI axial image. **(F)** ADC axial image.

At the 5th week, the patient had repeated fever (with the highest temperature of 39°C), increased sputum volume and indicated *Klebsiella pneumoniae* infection in the sputum culture. Considering that piperacillin tazobactam sodium was not effective against infection, meropenem (0.5 g) per 8 h was used to continue the anti-infection therapy. At this time, the patient underwent a re-examination of head MRI (Figure 2), which suggested brain parenchyma swelling in the left hippocampus that was considered to be caused by autoimmune encephalitis.

On day 38 of admission, the patient was given 0.4 g of cyclophosphamide twice per week. Following this, the patient presented symptoms of sinus sex tachycardia, gibberish, paroxysmal noisy and restraining attempt to sit up. The next day, efficacy of the dopamine and hydroxylamine drugs were gradually lowered and 5 mg of midodrine hydrochloride was added. After withdrawal of dopamine and serotonin in the afternoon, the patient's blood pressure became stabilized. As at this time, the patient's body temperature remained elevated, with the highest body temperature of 38.5°C. Serum procalcitonin (0.316 ng/mL) blood culture indicated no bacterial growth for 5 days, while sputum culture indicated *Klebsiella pneumoniae* infection. We suspected that the recurrent episodes of infection in the patient was associated with the immunosuppressive therapy.

On day 41 of hospitalization, the patient was treated with 0.4 g of cyclophosphamide for the third time. Since the patient's blood biochemistry indicated a significant reduction in albumin, we gave a temporary dose of human albumin (10 g). Although the highest temperature of the patient at the time was 38.7°C, the patient's mental symptoms was significantly better than before, except that the patient had occasional fidgety at night, which was almost completely normal at daytime and completely relevant to conversation with the others, without symptoms of epilepsy. Considering the patient's economic condition, exclusion examination of relevant tumor and re-examination of CSF antibody titer were not performed. The patient was discharged 3 days later and transferred to a local hospital for further hospitalization, despite the fact that the patient's infection was not fully corrected.

## Discussion

Autonomic dysfunction in autoimmune encephalitis usually includes sinus tachycardia, increased salivation, sinus bradycardia, hypotension, central fever, hypothermia and central hypoventilation, which are relatively common in NMDAR encephalitis (5, 6). This paper mainly discusses the refractory hypotension caused by autoimmune encephalitis. Prior to this, there is need to identify other related causes of hypotension such as septic shock, drug-induced hypotension and Neuroleptic Malignant Syndrome (NMS).

Septicaemia is usually defined as a life-threatening organ dysfunction that result from a host's dysfunctional response to infection (7). In 2016, the Third International Consensus Definition for Sepsis and Septic Shock (Sepsis-3) proposed that for the diagnosis of septic shock (even after being fully resuscitated), mean arterial pressure was required to maintain at ≥65 mmHg, with a serum lactic acid level of >2 mmol/L (18 mg/dL). According to this standard, it is possible to diagnose sepsis in patients during their hospitalization, but insufficient to fully explain that the persistent hypotension was due to sepsis. Apart from the persistent hypotension, no other septic shock-induced hypoperfusion was observed. Furthermore, when we administered piperacillin tazobactam sodium as the anti-infection treatment, the patient presented symptoms of infection and serum inflammatory indicators were significantly improved. We even stopped the antibiotics administration; however, the persistent hypotension still remained.

In addition to psychotropic drugs, we administered acyclovir, glycerol fructose injection and piracetam that may cause hypotension during the hospitalization. Acyclovir and glycerin fructose were discontinued prior to occurrence of the persistent hypotension. Piracetam was discontinued immediately after onset of the hypotension. Therefore, we did not consider the persistent hypotension in the patient to be drug-induced.

Many advanced autoimmune encephalitis symptoms clinically overlap with NMS. NMS is a rare but potentially fatal clinical syndrome characterized by altered mental status, delayed movement with rigidity and autonomic nervous dysfunction which as usually caused by the use of antipsychotics (8). Many patients with early undiagnosed autoimmune encephalitis are given antipsychotic drugs to help relieve the symptoms of acute agitation or delirium. This puts forward the question of whether refractory hypotension is a feature of autoimmune encephalitis itself or is the autonomic nervous dysfunction of NMS caused by these drugs.

Although our patient's presentation did match some characteristics of NMS, the diagnosis did not explain all the symptoms or their persistence. For instance, creatine kinase was elevated to 771.0 IU/L on day 2 of admission, which is four times higher than upper limit of the normal range. However, it may also be earlier associated with recurrent seizures in patients. Two weeks after olanzapine was discontinued, quetiapine fumarate was administered due to uncontrollable mental symptoms. As at this time, myosin of the patient was tested again and no increase was found. Considering that the seizures did not reoccur at this time, we suspected that the previous elevation of myosin was more correlated with seizures. According to the recommendations of the international multidisciplinary panel of experts for the diagnosis of NMS, administration of dopamine receptor antagonists or withdrawal of dopamine receptor agonists must be within 72 h of onset of the symptom. In this study, many of the presented symptoms began a few days before the psychotropic medications were administered and persisted for 72 h after they were discontinued.

Although no lesion was found by imaging, GABA receptor antibodies may affect the part of the central nervous system that controls blood pressure in a completely different way. This needs to be confirmed by larger sample size clinical studies in order to obtain more evidence to differentiate these cases from psychotropic drug-induced NMS. This may improve the understanding of both diseases. We hope that this case would arouse more attention and provide relevant reference for treatment of other similar patients.

## Data Availability Statement

The original contributions presented in the study are included in the article/supplementary material, further inquiries can be directed to the corresponding author/s.

## Ethics Statement

Written informed consent was obtained from the individual(s) for the publication of any potentially identifiable images or data included in this article.

## Author Contributions

ZS: put forward research ideas. WX: took the responsibility of communicating with the patient's family and obtaining the authorization in this paper. KH: responsible for drafting articles. All authors contributed to the article and approved the submitted version.

## Conflict of Interest

The authors declare that the research was conducted in the absence of any commercial or financial relationships that could be construed as a potential conflict of interest.
